# Coexistence of *Borrelia* spp. with different tick-borne pathogens in *Ixodes ricinus* ticks removed from humans in Poland

**DOI:** 10.1038/s41598-025-05885-2

**Published:** 2025-07-01

**Authors:** Julia Koczwarska, Justyna Polaczyk, Wiktoria Wieczorek, Olga Zdzienicka, Julia Żórańska, Agnieszka Pawełczyk, Renata Welc-Falęciak

**Affiliations:** 1 Department of Parasitology, Institute of Experimental Zoology, Faculty of Biology, University of Warsaw, ul. Miecznikowa 1, 02-096 Warsaw, Poland; 2 Department of Immunopathology of Infectious and Parasitic Diseases, Medical University of Warsaw, ul. Pawińskiego 3C, 02-106 Warsaw, Poland

**Keywords:** *Borrelia*, Tick-borne pathogens, Co-infection, *Ixodes ricinus*, Pathogen load, Droplet digital PCR, Parasitology, Pathogens

## Abstract

*Ixodes ricinus* tick is a primary vector of *Borrelia* spirochetes and various tick-borne pathogens in Europe. Multi-species infections are common among ticks, however, the mechanism by which *Borrelia* spp. coexists with other pathogens within the tick vector is poorly understood. Furthermore, the extent to which *Borrelia* spp. interact with other pathogens or how multi-species infections influence pathogen loads in ticks and consequently, their transmission success and pathogenicity, remains unclear. The aim of this study was to evaluate the impact of co-infections on occurrence and loads of *Borrelia* spp. and other pathogens in *I. ricinus*. In the years 2021–2022, we collected 2073 *I. ricinus* ticks from tick-bitten individuals from around Poland and analyzed individually for the presence of *Borrelia* spp., *Rickettsia* spp., *Neoehrlichia mikurensis*,* Anaplasma phagocytophilum*,* Babesia* spp. and *Bartonella* spp. using molecular methods. Loads of pathogens were determined with droplet digital PCR technique. Of the 324 ticks positive for *Borrelia* spp., 76 were co-infected with at least one different pathogen. We observed higher prevalence of *Babesia* spp. and *N. mikurensis* among *Borrelia* spp. – positive ticks than in ticks uninfected with *Borrelia* spp. (3.4% vs. 1.3% and 10.2% vs. 4.2% respectively). A similar positive correlation was observed between *Babesia* spp. and *N. mikurensis*. Additionally, the loads of *N. mikurensis* were nearly twice as high in *Babesia* spp. – positive ticks compared to those not infected with this pathogen. This study is among the first to explore influence of co-infections on pathogen loads in multi-infected ticks feeding on humans. Understanding the relationships between pathogens coexisting in ticks may broaden our insight into epidemiology of tick-borne diseases.

**Supplementary Information:**

The online version contains supplementary material available at 10.1038/s41598-025-05885-2.

## Introduction

Tick-borne diseases (TBDs) are considered a growing public health problem in Europe and the USA^1,2^. The most common TBD reported in Europe is Lyme borreliosis (LB), of which the number of cases has increased steadily since 1990, and more than 360,000 cases have been reported over two decades^3^. In Poland LB incidence rates almost doubled during a 10-year period from 33.2 in 2013 to 67.1 cases per 100,000 inhabitants in 2023^4,5^.

*Ixodes ricinus* tick is the main vector of *Borrelia burgdorferi* s.l. and a variety of other pathogenic microorganisms, including bacteria, viruses and piroplasms in Europe^3^. Due to

a combination of human behavior and extended tick activity related to global climate warming, the number of new cases of LB is expected to remain high and/or grow^6–8^. Moreover, other emerging TBPs including *Anaplasma phagocytophilum*, *Babesia* spp., *Borrelia miyamotoi*, *Neoehrlichia mikurensis* and spotted fever group *Rickettsia* spp. are becoming more prevalent and widespread in recent years^9,10^. Also, a role of ticks in the transmission of *Bartonella* spp.

is speculated^11^. Despite the confirmed pathogenicity of these microorganisms to humans, the incidence and severity of diseases caused by them are not fully investigated due to possible under-reporting associated with little awareness in clinical practice and/or lack of routine diagnostic modalities^6,12,13^.

According to multiple reports, ticks are commonly co-infected with two or more pathogenic species, with often high co-infection rates reaching up to even 65% in some regions^14–16^. It is widely accepted that reservoir hosts usually harbor a community of diverse pathogens and parasites including bacteria, protozoa, viruses, helminths and arthropods^17^. Multi-species infections in ticks are known to be linked to feeding on a large variety of animals, including birds and rodents, which exposes them to pathogens infecting these hosts^18^.

Co-infections in ticks create the risk of pathogen co-transmission to hosts, including humans^18^. It is especially relevant from the epidemiological point of view, considering that co-infections in vertebrate hosts are speculated to affect infectiousness of pathogens and susceptibility of hosts^19,20^. Moreover, according to many reports, co-infections in humans and animals can enhance disease severity (clinical symptoms, duration and intensity of infection) as well as complicate the diagnostic process and the TBD treatment^21–23^.

In ticks, new microbial interactions are being described, mainly between symbiotic bacteria and pathogens. The influence of gut endosymbionts on vector capacity by affecting pathogen colonisation has been described^9,24^. Shared environment for different pathogenic and parasitic species suggests possibility of both positive and negative interactions^19^. However, there is still limited data on the effect of co-infection with other pathogenic species on survival, colonization or transmission of TBPs in the arthropod vector. Also, the mechanism by which pathogens co-exist in ticks remains unexplored.

To date, few studies have been conducted determining the pathogen spread in ticks feeding on humans. Most studies have concentrated on questing ticks and ticks removed from animals, with nymphs and larvae usually being pooled, which did not provide full insight into the distribution of co-infections in ticks^15,25–31^. Assessing the occurrence of pathogens in ticks removed from the human skin provides precise information on the risk of human exposure to TBPs^32–34^. For determining the prevalence of *Borrelia* spp. in ticks, it is also important to consider that the abundance of spirochetes in questing ticks may be low and therefore often undetectable, while blood uptake triggers rapid bacteria proliferation after attachment, which increases its detectability in ticks removed from the skin^34,35^.

The purpose of this study was to (i) evaluate prevalence of TBPs in individual ticks removed from humans in Poland, (ii) compare the impact of co-infections on the prevalence of TBPs, (iii) to determine whether loads of TBPs in infected ticks depend on the co-infections with different pathogen species. Results obtained in this study will allow for a better insight into the relationships between pathogens co-existing in ticks. These interactions can potentially affect the ability of ticks to transmit pathogens or influence the success of pathogen transmission from tick to host, therefore, understanding the mechanisms by which pathogenic microorganisms and parasites coexist within ticks can in the future increase effectiveness of risk prediction of TBDs and opens the way for manipulating the tick microbiome to reduce pathogen transmission.

## Materials and methods

### Study design and ethics approval

The study reported here was conducted during a 2-year period. Ticks were collected throughout Poland from March to November of 2021 and 2022. The collected ticks originated from all 16 voivodeships in Poland in numbers ranging from 18 in Opolskie to 651 in Masovian voivodeship. Specimens were delivered directly by study participants or sent by a private delivery company to the Faculty of Biology, Department of Parasitology in tightly sealed containers filled with 70% ethanol within 5 days of removal from the skin by a physician or patients themselves. Information about our research was disseminated by the University of Warsaw website, websites dedicated to medicine, medical diagnostics and health care, local media, as well as distributed in the University community through email.

Each participant was included in the study after an informed consent and received information on the aims and the protocol of the study. For the participants under the legal age of consent (< 18 years old), one of the parents signed the agreement. The study protocol followed ethical guidelines of the 2013 Declaration of Helsinki and was approved by the Internal Review Board of the Warsaw Medical University (no. AKBE/73/2021). Written informed consent was obtained from all individual participants included in the study.

### Tick identification

Ticks were morphologically identified under stereoscopic microscope to species and developmental stage using a standard taxonomic key^36^. Specimens that were extensively damaged and therefore unidentifiable were not included in the study.

### DNA extraction and PCR analysis

For DNA isolation each tick was washed in 70% ethanol and sterile distilled water to avoid DNA contamination, and then homogenized with stainless steel beads using automatic Qiagen TissueLyser II (Qiagen, Hilden, Germany). Genomic DNA from adult, nymphal and larval ticks were isolated individually using DNeasy Blood & Tissue Kit (Qiagen, Hilden, Germany) according to the manufacturer’s protocol.

Genomic DNA was then used for molecular identification of the following pathogens: *A. phagocytophilum*, *Babesia* spp, *Bartonella* spp., *Borrelia* spp., *N. mikurensis* and *Rickettsia* spp. Standard PCR or nested PCR protocols were used for pathogen screening. Then PCR products were visualized on 1.5% agarose gels stained with Midori Green Stain (Nippon Genetics Europe, Germany), then analyzed in the Gel Doc XR+ (Bio-Rad, Hercules, California, USA). DNA of *A. phagocytophilum* was detected through amplification of the 546 bp fragment of 16 S rRNA using two sets of primers (initial reaction: ge3a, ge10r, nested PCR: ge9f, ge2) described in^37^. Screening for *Babesia* spp. in ticks was performed by amplification of the 559 bp 18 S rRNA fragment with GF2 and GR2 primers described in^38^. Detection of *Bartonella* spp. DNA was performed with PCR reaction using primers CS140f and BhCS1137n targeting the 1039 bp fragment of the enzyme citrate synthase gene (*gltA*)^39^. The 605-bp-long fragment of flagellin gene (*flaB*) specific to *Borrelia* spp. was detected by nested-PCR using two sets of primers: 132f, 905r for initial reaction and 220f, 824r for the nested reaction^40^. Screening for *N. mikurensis* in ticks was performed by amplification of the 654-bp-long fragment of the *groEL* gene with CNM_groEL_PCR_F and CNM_groEL_PCR_R primers reported in^29^. Primers CS409 and Rp1258 were used for the amplification of the 769 bp fragment of the enzyme citrate synthase gene (*gltA*) specific for *Rickettsia* spp^41^.

### RFLP analysis

Nested PCR-restriction fragment length polymorphism (RFLP) based on the *flaB* gene was used to differentiate *Borrelia* – positive isolates at the species level. Positive amplicons were digested with the restriction enzyme HpyF3I (Thermo Fisher Scientific, Waltham, MA USA), which recognizes the 5′C↓TNAG3′ sequence^42^ according to the manufacturer’s protocol. The digestion products were separated on a 2% agarose gel, then visualized and analyzed in the Gel Doc XR + imaging system (Bio-Rad, Hercules, California, USA). Randomly selected and inconclusive samples were sequenced using the Sanger method to confirm species identification.

### Sanger sequencing

TBP – positive amplicons from ticks were purified and sequenced in both directions by a private company (Nexbio Ltd., Lublin, Poland). Obtained nucleotide sequences were analyzed using BLAST NCBI and MEGA v. 11.0 software^43^ for sequence alignment and species typing using sequences deposited in GenBank of National Center for Biotechnology Information (NCBI)^44^. The new, representative nucleotide sequences of TBP species have been deposited in the GenBank database under accession numbers: PV124223, PV124288 – PV124290, PV173311, PV173333 – PV173342.

### Droplet digital PCR

In order to determine the concentrations of DNA targets of TBPs in individual nymphal and adult female ticks, absolute quantification with droplet digital PCR (ddPCR) was performed. Only intact ticks with a complete idiosome and gnathosome were included in those analyses.

*I. ricinus* extracts were quantified prior to ddPCR analysis using the NanoDrop One™ (Thermo Fisher Scientific, Waltham, MA) to determine the total concentration of double-stranded DNA present in each sample.

Quantification of TBP DNA targets in individual ticks was performed on the basis of the major surface protein 2 gene (*msp2*) for *A. phagocytophilum*^45^, the 18S rRNA gene for *Babesia* spp.^46^, the NADH dehydrogenase gamma-subunit gene (*nuoG)* for *Bartonella* spp.^47^, the 16S rRNA gene for *Borrelia* spp.^48^, the *groEL* gene for *N. mikurensis*^49^ and the enzyme citrate synthase gene (*gtlA*) for *Rickettsia* spp.^45^

ddPCR assay was performed in 22 µL reaction mixtures containing 11 µL of QX200™ ddPCR™ EvaGreen Supermix (Bio-Rad, Hercules, CA), forward and reverse primers at 100 nM each, 50 ng of template DNA and RNase-/DNase-free water on amount depending on the volume of the template DNA. Samples for which a concentration of 50ng DNA per reaction could not be obtained due to low or high total concentration of DNA were added at the maximum possible volume and diluted 10 times respectively. Afterwards, the obtained results were recalculated for concentration of 50 ng per reaction. Negative controls were performed in the absence of template DNA.

20 µL of ddPCR reaction mixture was loaded into an eight well DG8™ Cartridge (Bio-Rad) and droplets were formed with the Bio-Rad QX200™ Droplet Generator, following the manufacturer’s instructions. Generated droplets were then transferred to a 96-well plate and sealed with a Bio-Rad PX1™ PCR Plate Sealer, as recommended by the manufacturer. TBP DNA targets were amplified in a C1000 Touch™ Thermal Cycler using the following cycling conditions: an initial denaturation step at 95 °C for 5 min, followed by 40 cycles consisting of denaturation at 95 °C for 30 s and an annealing/extension step at 55 °C (for: *N. mikurensis* and *Rickettsia*) or 60 °C (for: *Borrelia* spp., A. *phagocytophilum*, *Babesia* spp., and *Bartonella* spp.) for 1 min, followed by signal stabilization steps at 4 °C and 90 °C for 5 min each and a 4 °C indefinite hold. The overall ramp rate was set at 2 °C/sec. After cycling, droplets were immediately analyzed on the QX200™ Droplet Reader. Bio-Rad QuantaSoft Analysis Pro software was utilized for absolute quantification to determine the concentration of target DNA copies in a sample (copies of template per µL of the final 1 × ddPCR reaction).

### PCR and droplet digital PCR controls

*Babesia microti* King’s College strain DNA isolated from infected BALB/c mice blood and sequenced *A. phagocytophilum*, *B. azfelii*, *B. taylorii*, *N. mikurensis* and *R. helvetica* DNA obtained from infected ticks, rodents and humans^32,50,51^ were used as positive PCR controls. Negative controls were performed in the absence of template DNA.

### Statistical analysis

Statistical analysis was performed using IBM SPSS Statistics v. 29 software (IBM Corp., Armonk, NY, USA). Prevalence of TBP infections (percentages of ticks infected) were analyzed using the maximum likelihood techniques based on loglinear analysis of contingency tables (HILOGLINEAR). For analysis of the prevalence of TBPs, we fitted the presence/absence of each pathogen as a binary factor (infected = 1, uninfected = 0) and then by year (2 levels: 2021 and 2022 for *A. phagocytophilum*, *Borrelia* spp., *Babesia* spp., *Bartonella* spp. and *Rickettsia* spp.), tick stadium (larvae, nymphs, adult ticks) and co-infection status for every each TBP (non-infected = 0, infected with second pathogen = 1). Values were reported with the 95% confidence intervals. P values < 0.05 were considered to be statistically significant.

For quantitative data regarding concentrations of TBPs DNA targets within individual ticks (the copies of template per µL of the final 1 × ddPCR reaction), a normality test was conducted with the Kolmogorov-Smirnov test. Than mean concentrations of DNA targets in ticks were compared against TBP co-infection status (non-infected = 0, infected with second pathogen = 1) with Mann-Whitney U-test. In order to compare target concentrations between more than two groups (*Borrelia* species), the Kruskal – Wallis H-test was performed. For both tests, the results for each group were summarized as mean values with the 95% confidence intervals. P values < 0.05 were considered to be statistically significant.

## Results

Total 2073 *Ixodes ricinus* ticks were collected during a 2-year period – 835 in 2021 and 1238 in 2022 respectively. Ticks, which were impossible to identify to a species level due to being extensively damaged, were excluded from the study. All 2073 ticks were identified to the developmental stage, of which 1447 (69.8%) were nymphs, 508 (24.5%) adult females, 29 (1.4%) adult males and 89 (4.3%) larvae. Owing to the small number of males, they were included in the analyses along with females as adult ticks. Merging sexes of ticks into one category did not have any significant effect on the statistical model, which was predominantly influenced by females.

### Prevalence of TBPs in *Ixodes ricinus* ticks

Detailed prevalence rates for each of the TBPs by year and tick developmental stage are summarized in Supplementary files 1 and 2 respectively.

### *Borrelia* spp.

**Fig. 1:**
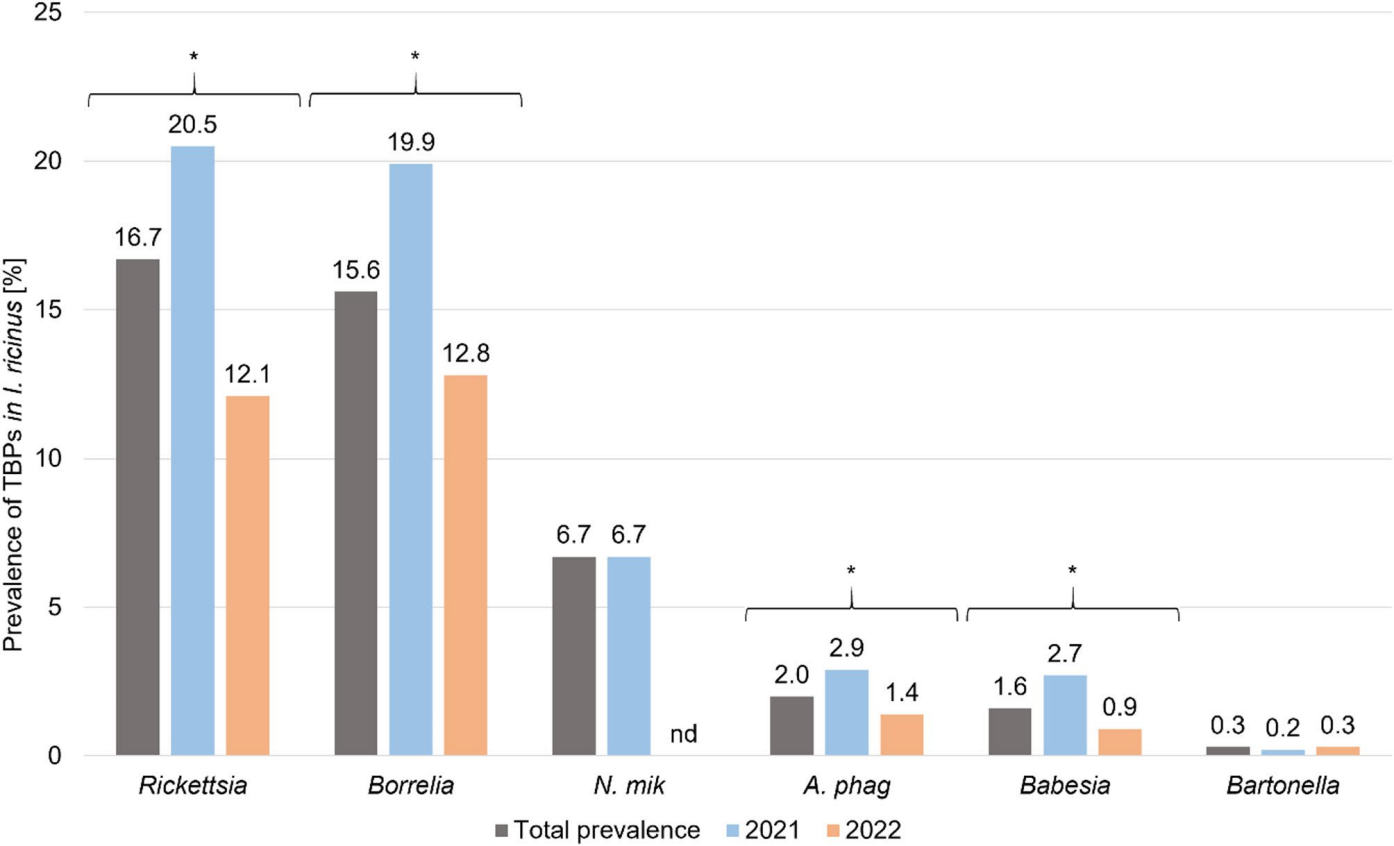
Prevalence of TBPs in individual *Ixodes ricinus* ticks removed from humans depending on the year of the study (2021–2022). *N* = 2073. For presence of *Neoehrlichia mikurensis* DNA only ticks collected in 2021 were tested (*N* = 832). *N. mik Neoehrlichia mikurensis*, *A. phag Anaplasma phagocytophilum*. Asterisk indicates *P* < 0.05 determined with maximum likelihood techniques based on loglinear analysis of contingency tables (HILOGLINEAR).

Overall 324/2073 (15.6%; 95% CI 14.1–17.2%) ticks were infected with *Borrelia* spp. *Borrelia* spp. prevalence was significantly higher in 2021 (166/835–19.9%; 17.3–22.7%) compared to 2022 (158/1238–12.8%; 95% CI 11.0–14.7%), (χ2 = 18.84, df = 1, *P* < 0.001) (Fig. 1). A significant effect of tick developmental stage on *Borrelia* spp. prevalence was also observed (χ2 = 23.45, df = 2, *P* < 0.001). Presence of *Borrelia* spp. DNA was detected in 3.4% larvae (3/89; 95% CI 1.0–8.7% ), 14.7% nymphs (212/1447; 95% CI 12.9–16.5%) and 20.3% adults (109/537; 95% CI 17.1–23.9%).

### *Rickettsia* spp.

*Rickettsia* spp. infection was detected in 347 of 2073 (16.7%; 95% CI 15.2–18.4%). Prevalence of *Rickettsia* spp. was significantly higher in 2021 compared to 2022 (196/835–23.5%; 95% CI 20.7–26.4% and 151/1238–12.2%; 95% CI 10.5–14.1% accordingly), (χ2 = 44.63, df = 1, *P* < 0.001) (Fig. 1). No effect of developmental stage was observed on *Rickettsia* spp. infection rate in ticks (χ2 = 5.39, df = 2, *P* = 0.068). Presence of *Rickettsia* DNA was detected in 11/89 (12.4%; 95% CI 6.7–20.4%) larvae, 230/1447 (15.9%; 95% CI 14.1–17.8%) nymphs and 106/537 (19.7%; 95% CI 16.5–23.3%) adult ticks.

### *Neoehrlichiamikurensis*

Only ticks collected in 2021 were tested for presence of *N. mikurensis* DNA resulting in prevalence 6.7% (56/832; 95% CI 5.2–8.6% ). No significant differences in *N. mikurensis* prevalence were observed between tick developmental stages (χ2 = 0.46, df = 2, *P* = 0.795). Presence of *N. mikurensis* DNA was found in 2/38 larvae (5.3%; CI: 1.1–15.8%), nymphs 35/542 (6.5%; 95% CI 4.6–8.8%) and 19/252 adult ticks (7.5%; 95% CI 4.8–11.3%).

### *Anaplasmaphagocytophilum*

Presence of *A. phagocytophilum* DNA was detected in 41/2073 of ticks (2.0%; 95% CI 1.4–2.6%). Infection rate of *A. phagocytophilum* in ticks was significantly higher in 2021 (24/835–2.9%; 95% CI 1.9–4.2%) compared to 2022 (17/1238–1.4%; 95% CI 0.8–2.1%), (χ2 = 5.65, df = 1, *P* = 0.017) (Fig. 1). *A. phagocytophilum* prevalence differed significantly between developmental stages and was higher in adult ticks (18/537–3.4%; 95% CI 2.1– 5.1%) than in nymphs (23/1447–1.6%; 95% CI 1.0–2.3%). No *A. phagocytophilum* DNA was detected in larvae (χ2 = 9.10, df = 2, *P* = 0.011).

### *Babesia* spp.

Overall 34/2073 ticks were infected with *Babesia* spp. (1.6%; 95% CI 1.2–2.3%) with significantly higher prevalence in 2021 (23/835–2.8%; 95% CI 1.8–4.0%) compared to 2022 (11/1238–0.9%; 95% CI 0.5–1.5%), (χ2 = 10.54, df = 1, *P* = 0.001) (Fig. 1). No significant differences in *Babesia* spp. prevalence were observed between tick developmental stages (χ2 = 3.23, df = 2, *P* = 0.198). Presence of *Babesia* spp. DNA was found in 1.8% nymphs (26/1447; 95% CI 1.2–2.6%) and 1.5% adult ticks (8/537; 95% CI 0.7–2.8%). No larvae infected with *Babesia* spp. were found (0/89).

### *Bartonella* spp.

Presence of *Bartonella* spp. DNA was detected only in 6/2073 (0.3%; 95% CI 0.1–0.6%) ticks. There were no significant differences in prevalence between 2021 (2/835–0.2%; 95% CI 0.0–0.8%) and 2022 (4/1238–0.3%; 95% CI 0.1–0.8%), (χ2 = 0.12, df = 1, *P* = 0.725) (Fig. 1). No effect of tick developmental stage on *Bartonella* spp. prevalence was observed (χ2 = 0.89, df = 2, *P* = 0.640). Most *Bartonella* spp. infections were detected in nymphs (5/2073–0.3%; 95% CI 0.1–0.8%), while *Bartonella* spp. DNA was found in only 1 adult tick (0.2%; 95% CI 0.0–0.9%) and none of larvae.

### Pathogen species distribution in ticks

**Fig. 2:**
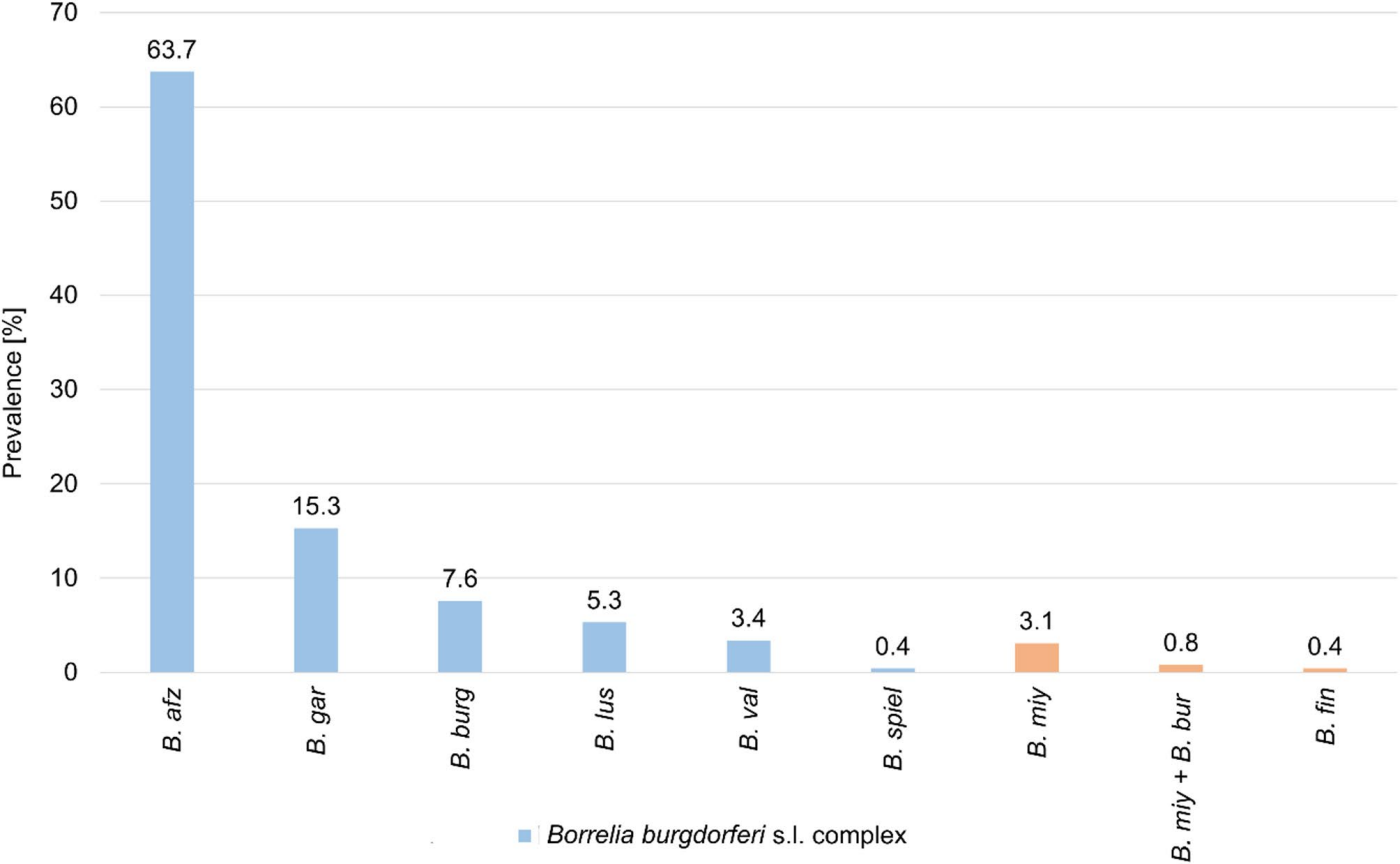
Prevalence of *Borrelia* species in *Ixodes ricinus* ticks removed from humans. *N* = 262 out of 326 *Borrelia* spp. – positive ticks. *B. afz Borrelia afzelii*,* B. gar Borrelia garinii*,* B. burg Borrelia burgdorferi* s.s., *B. lus Borrelia lusitaniae*,* B. val Borrelia valaisiana*,* B. spiel Borrelia spielmanii. B. miy Borrelia miyamotoi*,* B. fin Borrelia finlandensis*,* B. miy + B. bur co-infection of Borrelia miyamotoi and Borrelia burgdorferi* s.s.

Species typing of *Borrelia* spirochetes in infected ticks was performed using nested PCR-RFLP method based on the *flaB* gene or Sanger sequencing of 605 bp *flaB* gene PCR product. *Borrelia* species differentiation was successful for 262 of 326 *Borrelia* spp. – positive ticks (80.7%). The most frequently detected *Borrelia* species was *B. afzelii* (167/262–63.7%; 95% CI 57.8–69.4%) followed by *B. garinii* (40/262–15.3%; 95% CI 11.3–20.0%), *B. burgdorferi* s.s. (20/262–7.6%; 95% CI 4.9–11.3%), *B. lusitaniae* (14/262–5.3%; 95% CI 3.1–8.6%), *B. valaisiana* (9/262–3.4%; 95% CI 1.7–6.2%) and *B. spielmanii* (1/262–0.4%; 95% CI 0.0–1.8%), which all belong to *B. burgdorferi* s.l. complex. Additionally 8 of 262 ticks (3.1%; 95% CI 1.5–5.7%) were infected with *B. miyamotoi*, the causative agent of relapsing fever and 1/262 (0.4%; 95% CI 0.0–1.8%:) with *B. finladensis*, species of uncertain pathogenicity. 2 of 262 analyzed ticks (0.8%; CI: 0.2–2.4%) (two females) were co-infected with *B. miyamotoi* and *B. burgdorferi* s.s. (Fig. 2).

In order to confirm the specificity of PCR-screening, randomly selected amplicons of 546 bp fragment of 16 S rRNA for *A. phagocytophilum* and 654 bp fragment *groEL* gene for *N. mikurensis*, were sequenced using Sanger method. Genotyping resulted in all *A. phagocytophilum* isolates identical with *A. phagocytophilum* obtained from *I. ricinus* in Poland (KP245905)^45^ and all *N. mikurensis* isolates identical with *N. mikurensis* obtained from *I. ricinus* in Czech Republic (MN151367)^29^.

*Babesia* species differentiation was conducted on the basis of Sanger sequencing of the 559 bp 18 S rRNA fragment PCR product and was successful in 79.4% (27/34) of the *Babesia* spp.– positive tick samples. The most frequently detected *Babesia* species was *B. microti* (66.7% – 8/27) followed by *B. venatorum* (29.6%8/27). Only in 1/27 (3.7%) tick sample *B. canis* was detected resulting in an isolate identical with *B. canis* sequence obtained from *I. ricinus* from our previous studies in Poland (MW791420)^32^.

For *Rickettsia* spp., positive samples were randomly selected for genotyping and the PCR product of the 750 bp *gltA* fragment was sequenced using the Sanger method resulting in all amplicons identical with *R. helvetica* obtained from *I. ricinus* (MH018972)^52^ and *Dermacentor reticulatus* OQ689707^53^ from our previous studies in Poland.

All 6 PCR products positive for *Bartonella* spp. DNA were sequenced on the basis of 1039 bp fragment of *gltA* gene with Sanger method resulting in all sequences identical to *B. taylorii* isolate obtained from *Microtus oeconomus* in Poland (EU014274)^39^.

### Co-infections in *Ixodes ricinus* ticks

596 of 2073 ticks (28.8%) were infected with at least one pathogen – 490 of infected ticks (82.2%) harbored a single agent, while 96 (16.1%) and 10 (1.7%) were co-infected with two and three pathogens respectively. Of 324 ticks infected with *Borrelia* spp., 76 (23.5%) were co-infected with one different pathogen species and 10 (3.1%) with two different pathogen species (including two females triple-infected with *Rickettsia* spp. and two different *Borrelia* species: *B. miyamotoi* and *B. burgdorferi* s.s.). The most common co-infections in ticks removed from humans were *B. afzelii* with *Rickettsia* spp. (25 specimens: 17 nymphs, 8 females) followed by *B. garinii* with *Rickettsia* spp. (10 specimens: 8 nymphs and 2 females) and *B. afzelii* with *N. mikurensis* (9 specimens: 1 larva, 5 nymphs, 2 females and 1 male). Detailed dual and triple co-infection distribution in tested ticks are shown in the Supplementary files 3 and 4, respectively. Table 1 provides P-values obtained for associations between TBPs prevalence in co-infected ticks.

**Table 1:** P values obtained for associations between TBPs prevalence in co-infected ticks removed from humans in poland.

	*Borrelia*	*A. phag*	*Babesia*	*Bartonella*	*N. mik*	*Rickettsia*
*Borrelia*	–	0.528	0.015*	0.284	0.046*	0.444
*A.phag*		–	0.242	0.624	0.065	0.638
*Babesia*			–	0.083	0.017*	0.745
*Bartonella*				–	0.597	0.138
*N.mik*					–	0.155
*Rickettsia*						–

*Borrelia Borrelia* spp., *A. phag Anaplasma phagocytophilum*, *Babesia Babesia* spp., *N. mik Neoehrlichia mikurensis*, *Rickettsia – Rickettsia* spp.

Asterisk indicates *P* < 0.05 determined with maximum likelihood techniques based on loglinear analysis of contingency tables (HILOGLINEAR).

### Co-infections with *Borrelia* spp.

**Fig. 3:**
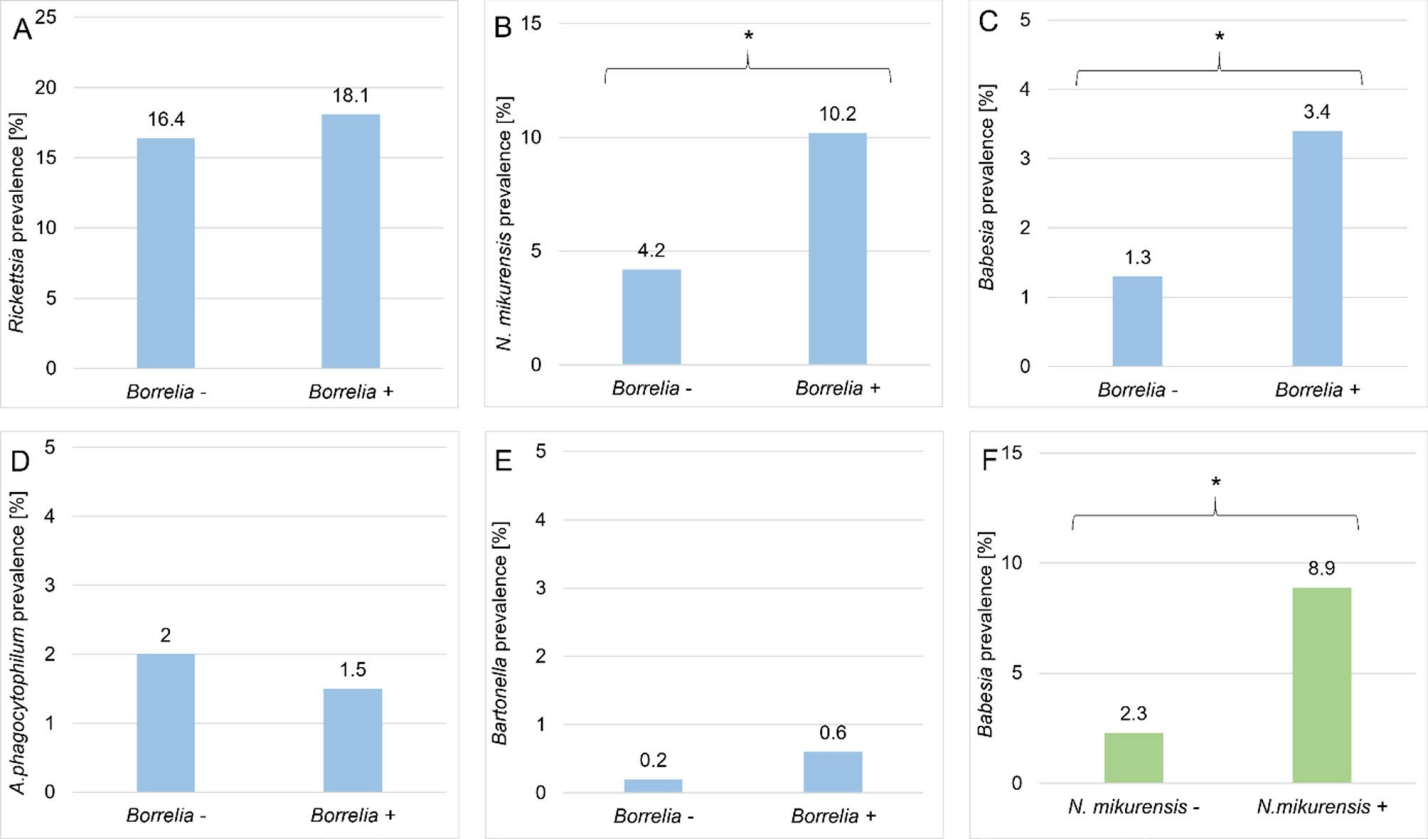
Prevalence of TBPs in single-infected and co-infected ticks. (**A**) prevalence of *Rickettsia* spp. in *Borrelia* spp. – and *Borrelia* spp. + ticks, (**B**) prevalence of *Neoehrlichia mikurensis* in *Borrelia* spp. – and *Borrelia* spp. + ticks, (**C**) prevalence of *Babesia* spp. in *Borrelia* spp. – and *Borrelia* spp. + ticks, (**D**) prevalence of *Anaplasma phagocytophilum* in *Borrelia* spp. – and *Borrelia* spp. + ticks, (**E**) prevalence of *Bartonella* spp. in *Borrelia* spp. – and *Borrelia* spp. + ticks, (**F**) prevalence of *Babesia* spp. in *Neoehrlichia mikurensis* – and *Neoehrlichia mikurensis* + ticks. Asterisk indicates *P* < 0.05 determined with maximum likelihood techniques based on loglinear analysis of contingency tables (HILOGLINEAR).

The most frequent co-infection was *Borrelia* spp. with *Rickettsia* spp. and was detected in 59/2073 (2.8%; 95% CI 2.2–3.6%) ticks. Nevertheless, no effect of *Borrelia* spp. was observed on *Rickettsia* spp. infection rate (χ2 = 0.59, df = 1, *P* = 0.444). *Rickettsia* spp. – positive ticks were observed in similar frequency in both *Borrelia* spp. – positive (59/326–18.2%; 95% CI 14.3–22.7%) and *Borrelia* spp. – negative ticks (288/1747–16.5%; 95% CI 14.8 – 18.3%) (Fig. 3A).

17/832 (2.0%; 95% CI 1.2–3.2%) ticks were co-infected with *Borrelia* spp. and *N. mikurensis*. Statistical analysis of co-infection in ticks showed significant differences of *N. mikurensis* infection rate in positive and negative for *Borrelia* spp. ticks χ2 = 3.98, df = 1, *P* = 0.046). Prevalence of *N. mikurensis* was higher in *Borrelia* spp. – infected ticks (17/160–10.4%; 95% CI 6.4–15.8%) compared to ticks uninfected with *Borrelia* spp. (39/672–5.8%; 95% CI 4.2–7.7%) (Fig. 3B).

11/2073 ticks (0.5%; 95% CI 0.3–0.9%) were co-infected with *Borrelia* spp. and *Babesia* spp. *Babesia* spp. infection was more frequently detected in *Borrelia* spp. – positive ticks (11/326–3.4%; CI: 1.8–5.8%) than ticks uninfected with *Borrelia* spp. (23/1747–1.3%; CI: 0.9–1.9%). These differences were confirmed to be statistically significant (χ2 = 5.97, df = 1, *P* = 0.015) (Fig. 3C).

*Borrelia* spp. and *A. phagocytophilum* co-infection was detected in 5/2073 (0.2%; 95% CI 0.1–0.5%) ticks. No significant differences in *A. phagocytophilum* prevalence was found between *Borrelia* spp. – positive (5/326–1.5%; 95% CI 0.6–3.3%) and *Borrelia* spp. – negative ticks (36/1747–2.1%; CI: 1.5–2.8%), (χ2 = 0.40, df = 1, *P* = 0.528) (Fig. 3D).

In 2/2073 ticks (0.1%; 95% CI 0.02–0.3%) co-infection with *Borrelia* spp. and *Bartonella* spp. was detected. Prevalence of *Bartonella* spp. in *Borrelia* spp. – positive and *Borrelia* spp. – negative ticks did not differ significantly and was 0.6% (2/236; CI: 0.1–2.0%) and 0.2% (4/1747; CI: 0.1–0.5%) respectively (χ2 = 1.15, df = 1, *P* = 0.284) (Fig. 3E).

### Co-infections with other TBPs

*A. phagocytophilum* and *Rickettsia* spp. co-infection was detected in 8/2073 (0.4%; 95% CI 0.2–0.7%) ticks. *A. phagocytophilum* prevalence was slightly higher in *Rickettsia* spp. – negative ticks (33/1726–1.9%; CI: 1.3–2.6%) compared *Rickettsia* spp. – positive (8/347–2.3%; 95% CI 1.1–4.3%), but without statistically significant differences (χ2 = 0.22, df = 1, *P* = 0.638).

5 of 2073 ticks (0.2%; 95% CI 0.1–0.5%) were found to be co-infected with *Rickettsia* spp.and *Babesia* spp. No effect of *Babesia* spp. on *Rickettsia* spp. prevalence was observed. Ticks infected with *Babesia* spp. were observed in similar frequency in both *Rickettsia* spp. – positive (5/347–1.4%; 95% CI 0.6 – 3.1%), and *Rickettsia* spp. – negative ticks (29/1726–1.7%; 95% CI 1.2–2.4%), (χ2 = 0.106, df = 1, *P* = 0.745).

No co-infection of *Rickettsia* spp. and *Bartonella* spp. was detected in examined ticks. *Bartonella* spp. was detected only in 6/1726 (0.3%; 95% CI 0.1–0.7%) *Rickettsia* – negative ticks (χ2 = 2.20, df = 1, *P* = 0.138).

9/832 ticks (1.1%; CI: 0.5–2.0%) were co-infected with *Rickettsia* spp. and *N. mikurensis.* Prevalence of *Rickettsia* spp. was lower in ticks infected with *N. mikurensis* (9/56–16.1%; 95% CI 8.3–27.3%) compared to tick uninfected with this pathogen (187/776–24.1%; CI: 21.2–27.2%), but without statistically significant differences (χ2 = 2.02 df = 1, *P* = 0.155).

No co-infection of *A. phagocytophilum* and *Babesia* spp. was detected in examined ticks. *Babesia* spp. was found only in 34/2032 (1.7%; 95% CI 1.2–2.3%) *A. phagocytophilum* – negative ticks (χ2 = 1.37, df = 1, *P* = 0.242).

No co-infection of *A. phagocytophilum* and *N. mikurensis* was detected in examined ticks. *A. phagocytophilum* was found only in 24/776 (3.1%; 95% CI 2.0–4.5%) *N. mikurensis* – negative ticks (χ2 = 3.40, df = 1 *P* = 0.065).

No co-infection of *A. phagocytophilum* and *Bartonella* spp. was detected in examined ticks. *Bartonella* spp. was found only in 6/2032 (0.3%; 95% CI 0.1–0.6%) *A. phagocytophilum* – negative ticks (χ2 = 0.24, df = 1, *P* = 0.624).

5/832 (0.6%; 95% CI 0.2–1.3%) ticks were found to be co-infected with *Babesia* spp. and *N. mikurensis*. Prevalence of *Babesia* spp. was significantly higher in ticks infected with *N. mikurensis* (5/56–8.9%; 95% CI 3.5–18.5%) than ticks uninfected with this pathogen (18/776–2.3%; 95% CI 1.4–3.6%), (χ2 = 5.65, df = 1, *P* = 0.017) (Fig. 3F).

*Babesia* spp. and *Bartonella* spp. co-infection was detected in only 1/2073 (0.05%; 95% CI 0.01–0.2%) ticks. *Bartonella* prevalence was higher in *Babesia* spp. – positive ticks (1/34–2.9%; CI: 0.3–12.9%) compared *Babesia* spp. – negative ticks (5/2039–0.2%; 95% CI 0.1–0.5%), but without statistically significant differences (χ2 = 3.00, df = 1, *P* = 0.083).

No co-infection of *N. mikurensis* and *Bartonella* spp. was found in *I. ricinus* ticks. *Bartonella* spp. DNA was found only in 2/776 ticks tested negative for *N. mikurensis* (0.3%; 95% CI 0.1–0.8%), (χ2 = 0.28, df = 1, *P* = 0.597).

### Pathogen loads – droplet digital PCR

#### Differences between *Borrelia* species

**Fig. 4:**
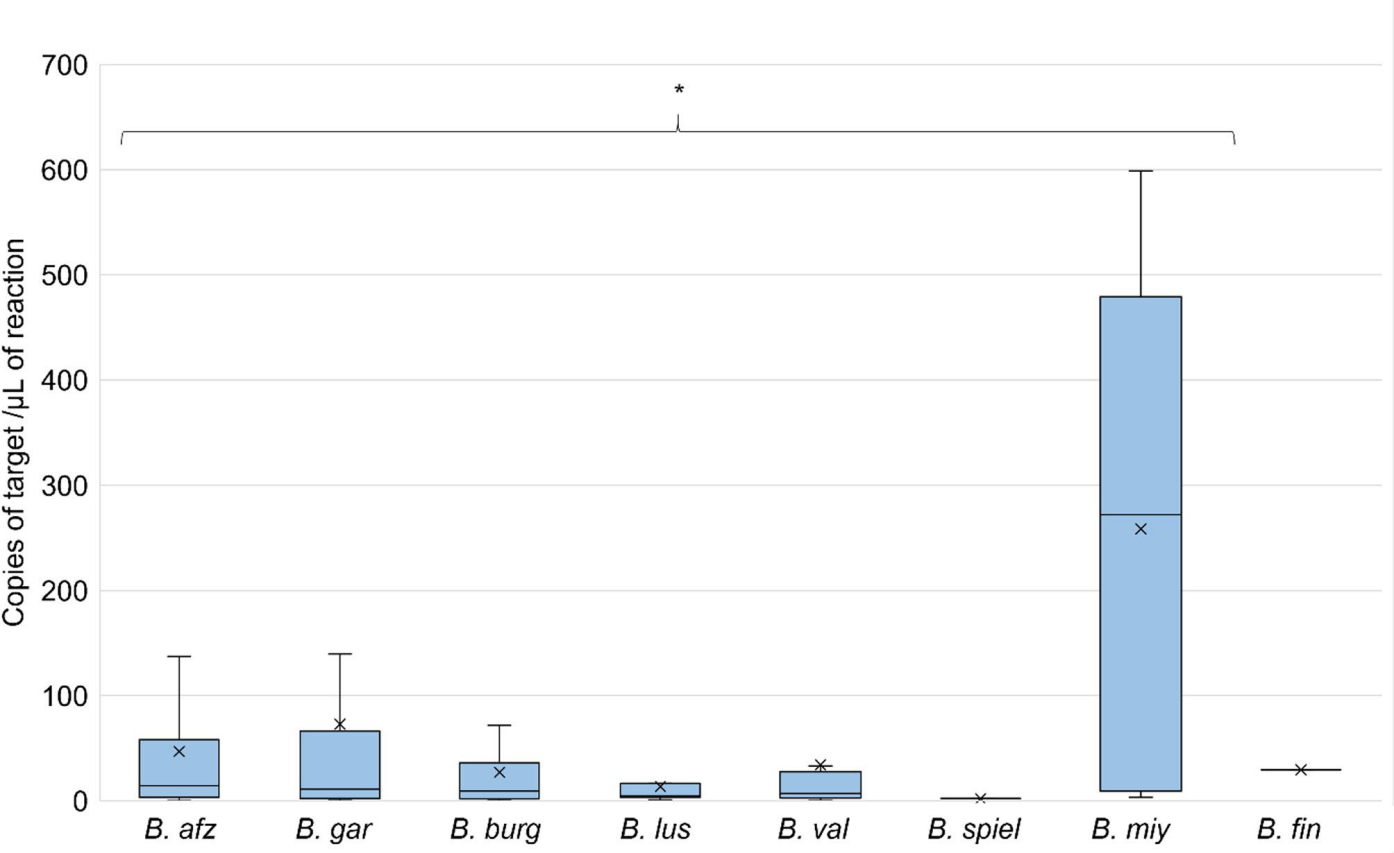
Loads of spirochetes in individual ticks infected with different *Borrelia* species determined by droplet digital PCR. *N* = 246 out of 326 *Borrelia* spp. – positive ticks. *B. afz Borrelia afzelii*,* B. gar Borrelia garinii*,* B. burg Borrelia burgdorferi* s.s., *B. lus Borrelia lusitaniae*,* B. val Borrelia valaisiana*,* B. spiel Borrelia spielmanii*,* B. fin Borrelia finlandensis*,* B. miy Borrelia miyamotoi.* The concentrations of target DNA copies in ticks are presented as copies of template per µL of the final 1 × ddPCR reaction. Ends of boxes represent the 25th and 75th percentiles with medians marked as horizontal lines and means as crosess. Vertical whiskers represent 10th and 90th percentiles. An asterisk indicates a *P* < 0.05 determined by Mann-Whitney U-test in comparision with the mean value obtained for *B. burgdorferi* s.l. complex.

The mean numbers of *Borrelia* spp. 16S rRNA targets did not differ significantly within *B. burgdorferi* s.l. complex and were as follows: *B. afzelii* – (46.8; 95% CI 32.8–60.9), *B. garinii* – (72.9; 95% CI 23.2–122.6), *B. burgdorferi* s.s. – (26.8; 95% CI 7.8–45.9), *B. lusitaniae* – (13.5; 95% CI 3.7–23.3), *B. valaisiana* – (34.2; 95% CI -18.9–87.2), (Kruskal-Wallis H-test, H = 11.16, df = 8, *P* = 0.193). Only one tick was quantified for *B. spielmanii*, resulting in 1.9 copies of the target. Additionally one tick was infected with *B. finlandensis*, species of uncertain pathogenicity, with number of targets of 29.3 (Fig. 4). Interestingly the mean numbers of *Borrelia* spp. 16S rRNA targets were four times higher in ticks infected with *B. miyamotoi* (258.5; 95% CI 51.9–465.2) comparing to *Borrelia burgdorferi* s.l. *–* infected ticks (47.1; 95% CI 34.7–59.5). This difference was statistically significant (Mann-Whitney U-test, Z = -2.35, *P* = 0.008) (Fig. 4). Due to this difference in target numbers and not certain pathogenicity of *B. finlandensis*, for further analysis we decided to include only *B. burgdorferi* s.l. (*B. afzelii*,* B. garinii*,* B. burgdorferi* s.s., *B. lusitaniae*,* B. valaisiana*,* B. spielmanii.* We excluded ticks infected with unknown *Borrelia* species and coinfected with two different *Borrelia* species.

#### Co-infections with *Borrelia* spp. and other TBPs

The detailed results of the pathogen load analyses, including the number of tested ticks, are provided in Supplementary file 5.

**Fig. 5:**
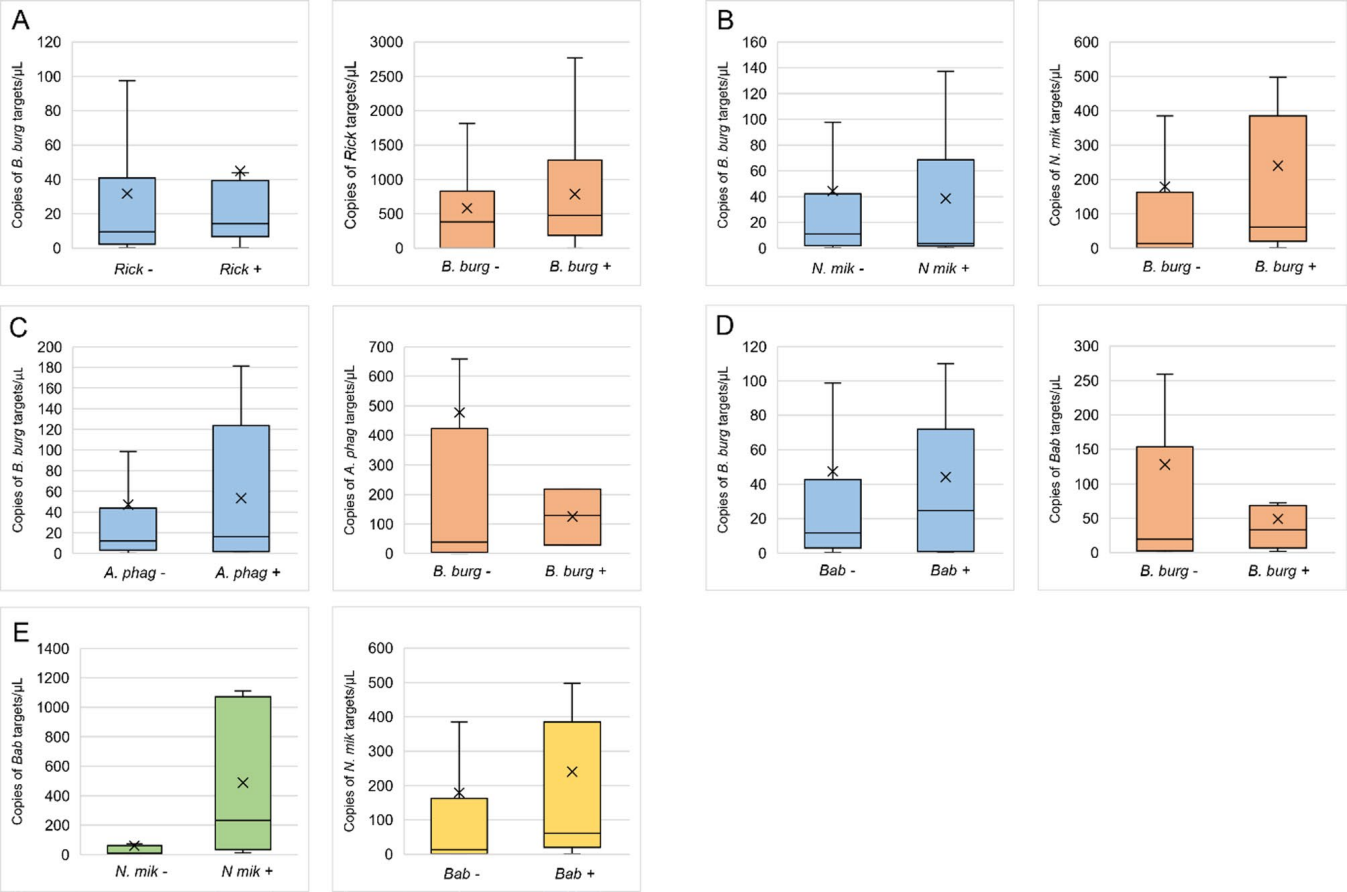
Loads of selected TBPs depending on presence/absence of other TBP in ticks determined by droplet digital PCR. *B. burg Borrelia burgdorferi* s.l., *Rick Rickettsia* spp., *N. mik Neoehrlichia mikurensis*,* A. phag Anaplasma phagocytophilum*,* Bab Babesia* spp. The concentrations of target DNA copies in ticks are presented as copies of template per µL of the final 1 × ddPCR reaction. Ends of boxes represent the 25th and 75th percentiles with medians marked as horizontal lines and means as crosess. Vertical whiskers represent 10th and 90th percentiless. An asterisk indicates a *P* < 0.05 determined by Mann-Whitney U-test.

The mean numbers of *B. burgdorferi* s.l. 16S rRNA targets were slightly higher in *Rickettsia* spp. – positive ticks compared to *Rickettsia* spp. – negative (44.8; 95% CI 25.3–64.4 and 31.7; 95% CI 24.5–38.9 respectively), but without statistically significant differences (Mann-Whitney U-test, Z = 1.62, *P* = 0.106). Similarly the higher mean numbers of *Rickettsia* spp. *gtlA* targets were found in *Borrelia* spp. – positive ticks than in *Borrelia* spp. – negative ticks (784.3; 95% CI 429.5–1139.0 and 581.6; 95% CI 310.5–852.6), but also without statistical significance (Mann-Whitney U-test, Z = -1.07, *P* = 0.283) (Fig. 5A).

The mean numbers of *B. burgdorferi* s.l. 16S rRNA targets were slightly lower in *N. mikurensis* – positive ticks (38.5; 95% CI 1.3–75.8) compared to *N. mikurensis* – negative (44.2; 95% CI 25.2–63.3), but without statistically significant differences (Mann-Whitney U-test, Z = -0.15, *P* = 0.884). In contrast, the mean numbers of *N. mikurensis groEL* targets were higher in *Borrelia* spp. – infected ticks than in ticks negative for this pathogen (239.9; 95% CI -43.6–523.5 and 179.0; 95% CI 39.5–318.4 respectively), but also without statistical significance (Mann-Whitney U-test, Z = -1.31, *P* = 0.190) (Fig. 5B).

The mean numbers of *B. burgdorferi* s.l. 16S rRNA targets were similar in ticks infected with *A. phagocytophilum* (53.4; 95% CI -41.2–148.1) and non-infected with this pathogen (47.2; 95% CI 34.3–60.2), (Mann-Whitney U-test, Z = -0.25, *P* = 0.801). For *A. phagocytophilum* the mean numbers of *msp2* targets were lower in *Borrelia* spp. – infected ticks (124.9; 95% CI 95% -110.4–360.2) compared to *Borrelia* spp. – negative ticks (476.8; 95% CI 96.4–857.3), but without statistically significance (Mann-Whitney U-test, Z = -046, *P* = 0.643) (Fig. 5C).

The means of *B. burgdorferi* s.l. 16S rRNA targets were similar in both *Babesia* spp. – positive and *Babesia* spp. – negative ticks (44.2; 95% CI 6.5–81.9 and 47.5; 95% CI 34.2–60.8 respectively), (Mann-Whitney U-test, Z = -0.32, *P* = 0.749). The mean numbers of *Babesia* spp. 18S rRNA targets however were lower in *Borrelia* spp.– positive ticks (48.9; 95% CI -0.1–97.9) than in ticks negative for *Borrelia* spp. (128.1; 95% CI 28.6–227.6), but the result was not statistically significant (Mann-Whitney U-test, Z = -0.05, *P* = 0.961) (Fig. 5D).

The ﻿numbers of *Bartonella* spp. *nuoG* targets in all tick samples were low (> 2 copies of template per µL of the final 1 × ddPCR reaction). Combined with the small number of samples obtained, it was not possible to perform reliable quantitative analyses.

The assessment of the pathogen loads in co-infections with other TBPs was performed only for *Babesia* spp. and *N. mikurensis*, as a positive correlation regarding prevalence was found between these pathogens. The mean numbers of *N. mikurensis groEL* targets were almost twice higher in *Babesia* spp. – infected ticks compared to *Babesia* spp. – negative ticks (330.1; 95% CI -192.3–853.5 and 188.6; 95% CI 52.5–324.8), however these differences were not statistically significant (Mann-Whitney U-test, Z = 1.35, *P* = 0.178). Likewise mean numbers of *Babesia* spp. 18S rRNA targets were higher in *N. mikurensis* – positive ticks than in ticks negative for this pathogen (488.7; 95% CI -181.9–1159.2 and 60.5; 95% CI -12.0–133.0). This correlation was found to be statistically significant (Mann-Whitney U-test, Z = -2.22, *P* = 0.026) (Fig. 5E).

## Discussion

Here we performed a detailed analysis of the TBP occurence and inter-pathogen associations in ticks removed from humans from Poland. The implementation of the novel droplet digital PCR method allowed us to perform more accurate analyses considering the possible influence of co-infection not only on the prevalence of pathogens, but also on their quantity, which may be reflected in the ability of these microorganisms to replicate and colonize ticks, and consequently on the possibility of their transmission from tick to host. Results obtained in our study can benefit public health through a better understanding of the interactions between human pathogenic microorganisms in these important arthropod vectors.

### Prevalence of TBPs

The most prevalent TBP in *I. ricinus* collected from humans in our study was *Rickettsia* spp. followed by *Borrelia* spp. and *N. mikurensis*. Similar results were obtained in a study conducted in the Netherlands, where authors found *Rickettsia* spp., *Borrelia burgdorferi* s.l. and *N. mikurensis* in 20%, 13% and 7% of questing nymphs respectively^6^. In a lower number of ticks we detected DNA specific to *A. phagocytophilum*, *Babesia* spp. and *Bartonella* spp. (2.0%, 1.6% and 0.3% respectively).

Overall *Rickettsia* spp. prevalence determined in our study was similar to results obtained formerly in Poland (18.7%)^25^ and higher compared to results from our previous studies conducted on questing ticks (5.6%)^52^. However, there are reports of higher rates of infected ticks in Poland reaching up to 42.3%^54^. The percentages of *Rickettsia* spp. – infected ticks in Europe, likewise in Poland, vary strongly depending on the region, from as low as 4.5% reported in Austerlitz, the Netherlands^55^to even 63% noted in Hamburg, Germany^56^.

Overall *Borrelia* spp. prevalence in ticks was lower than in our previous study (25.3%)^32^. However, *Borrelia* spp. infection rates in *I. ricinus* populations vary significantly depending on location. In a review concerning green urban areas in 24 European countries, the prevalence of spirochetes in *I. ricinus* ranged from 3.1 to 38.1% with overall percentage of infected ticks similar to detected in our study (17.3%)^57^.

Prevalence of *N. mikurensis* obtained in our study was in accordance with average infection rate in questing *I. ricinus* of 6–8% reported in review covering 16 European countries^6,58^. However, the prevalence of this pathogen in our study was higher than that found in previous years in Poland. Among a few reports in this country, the percentage of infected ticks was found at the levels of 0.5% in 2014 reported by Welc-Falęciak et al.^50^2.8% in 2010–2016 declared by Sawczyn-Domańska et al.^26^ and 2.9% in 2012–2015 reported by Kowalec et al.^52^. Whereas in a research carried out in 2021 in North-Eastern Poland, the percentage of *I. ricinus* infected with this pathogen was found to be 14.5% and 13.3% in ticks collected from vegetation and dogs, respectively^59^which is about twice as high as in our studies.

*N. mikurensis* is considered an emerging tick-borne disease in Europe, and it seems that the number of infected ticks has been increasing in recent years in Poland^59^. However, due to the small number of reports, further screening of this pathogen is necessary.

Infection rate of *A. phagocytophilum* in ticks was similar as obtained in a previous study conducted on ticks in natural forests in Poland (1.7%)^50^ and slightly lower compared to the former study conducted on questing ticks in urban and rural areas (5.8%)^52^. In a review concerning TBPs in *I. ricinus* from different European countries, estimated prevalences varied from 0.4% in Murbach, France, to 2.1% in Austerlitz, The Netherlands, and as high as 11.9% in Grib Skov, Denmark^55^.

Percentage of ticks tested positive for *Babesia* spp. DNA was consistent with 1.3% prevalence obtained in our previous studies conducted in 2016–2019 in Poland^32^. This result seems to be also in accordance with the global infection rate of zoonotic *Babesia* species in tick vectors, which ranges locally from 0.7–3.7%^60^.

In our study, we obtained a low rate of *Bartonella* spp. infection in ticks. In experimental studies, the competence of *I. ricinus* ticks as a vector of this pathogen has been proven^61^. However, the actual importance of *I. ricinus* in the circulation of this pathogen under natural conditions is still not confirmed^62^. Among the few reports regarding the prevalence of *Bartonella* in this tick species, many failed to detect this pathogen^31,55,62–64^. Although in a few studies conducted in Europe, infection of *Bartonella* spp. in *I. ricinus* was reported with prevalences equal to 0.1% in Wasselone, France^55^, 1.7% and 4.8% in Poland^65,66^ and 2.1% in Austria^11^.

### Differences between tick developmental stages

We found a significant effect of developmental stage on prevalence for two pathogens: *Borrelia* spp. and *A. phagocytophilum.* For both pathogens, infections rates increased with every subsequent tick developmental stage. The reason for these inter-stage differences is probably acquiring pathogens on the path of the life-cycle of ticks. For most of *Borrelia* species, transovarial transmission is marginal and ticks get infected through feeding on various hosts^23,67^. As ticks feed only once in every developmental stage, the possibility to acquire pathogens increases with every bloodmeal. Similarly, the probability of infection with *A. phagocytophilum* rises with every developmental stage, and is highest in adult ticks, as ungulates on which nymphs can feed are the most important hosts to this species^60^. For *Rickettsia* spp. infection rates were similar in all developmental stages of ticks, which is probably a result of transovarial transmission, as this pathogen is considered a tick endosymbiont^6,52^.

### Annual fluctuations in TBP prevalence

Interestingly, we observed a significant decrease in annual prevalence of four TBPs between 2021 and 2022. It appears that proportions of *I. ricinus* ticks infected with some TBPs tend to fluctuate over the years as we found similar inter-annual changes in *Borrelia* spp. prevalence in our previous studies, where we reported a decrease of *Borrelia* spp. infection rate from 38% in 2016 to 25% in 2019^32^. Annual and seasonal prevalence changes were also noted for *Rickettsiales* (*A. phagocytophilum*,* N. mikurensis and Rickettsia* spp.) in another study^52^. We suspect that these differences may be caused primarily by climatic and ecological factors that may affect tick abundance, availability of reservoir hosts, primarily rodents and birds, which in turn affect the level of tick TBP infestation^68^. In case of *Rickettsia* spp., which is considered a tick endosymbiont and is transmitted transovarially, the causes of inter-annual differences are still unknown. There is a speculation that temporal variations in gut microbiota of ticks may be linked to differences in the composition of the environmental microbiota as well as abiotic factors that can directly affect life-cycle and distribution of ticks such as temperature, humidity and rainfall^54,69^.

### TBPs species

In our study we found 6 genospecies of *B. burgdorferi* s.l. complex in ticks, from which the most commonly found were *B. afzelii* followed by *B. garinii* and *B. burgdorferi* s.s. The less frequently detected species were *B. lusitaniae*, *B. valaisiana* and *B. spielmanii*. Additionally, we reported occurrence of *B. miyamotoi*, the causative agent of relapsing fever, in tested ticks. The same *Borrelia* species composition was found in ticks removed from humans in Poland previously, with slight differences in prevalences between pathogen species depending on the year and higher overall prevalence of *B. miyamotoi* compared to this study (8.4%)^32^. Moreover, one tick was tested positive for *B. finlandensis*, a species of uncertain pathogenicity, which was also detected previously in Poland before^70^.

We found two female ticks co-infected with two *Borrelia* species, mainly *B. miyamotoi* and *B. burgdorferi* s.s. The most possible explanation of this co-infection, as both ticks were adults, is that they acquired these pathogens during feeding on reservoir hosts, and in case of *B. miyamotoi* transovarial transmission was also possible^67,71^.

Interestingly, we found that despite quite low prevalence of *B. miyamotoi* in ticks, loads of this pathogen in individual ticks were much higher compared to other *Borrelia* species. Pathogen loads within *B. burgdorferi* s.l. complex were on the other hand similar. Comparable results were obtained in another study, where authors found significantly higher pathogen loads in *B. miyamotoi* compared to *B. afzelii*, *B. garinii* and *B. burgdorferi* s.s. and no differences between other *Borrelia* species^34^. Higher loads of *B. miyamotoi* compared to other *Borrelia* species were also detected recently in *I. persulcatus* in Mongolia^71^. The exact reason for this pattern is unknown. However, possible causes are speculated to be: (i) obtaining higher loads of *B. miyamotoi* during bloodmeal, as reservoir hosts are proven to harbor higher loads of this pathogen in their blood than other *Borrelia* species, (ii) possible high survival rate of *B. miyamotoi* during the tick molting process^34^. It is worth noting that higher loads of *B. miyamotoi* may reflect greater transmission success of this pathogen, however, further research in this area is needed^34^.

The most frequently detected *Babesia* species was *B. microti* followed by *B. venatorum*. Only in one tick sample *B. canis* was detected. All three species were reported in *I. ricinus* removed from humans in our previous study, with similar infection rates^32^.

### Co-infections—differences in TBP prevalence and loads

Coexistence of different TBPs in ticks is widely reported in not only Europe, but also the USA and Asia^23^. Ticks acquire further pathogens by feeding on infected reservoir hosts, contracting pathogens from other ticks via cofeeding, interrupted blood meals and, for some pathogens, through transovarian transmission^17,32,72^.

In our study, we found a significant correlation between *Babesia* spp. and *Borrelia* spp. in tested ticks. Infection rates of *Babesia* spp. were more than twice as high in *Borrelia* spp. – infected ticks compared to ticks free of this pathogen. Similar results were obtained in our previous studies with *Babesia* spp. infection rates equal to 2.7% and 0.8% for *Borrelia* spp. – positive and *Borrelia* spp. – negative ticks respectively^32^. The co-occurrence of these pathogens in ticks is not surprising, as the dominant species involved in co-infections (*B. afzelii* and *B. microti*) share a common host reservoir, which are mainly rodents, which creates conditions for co-transmission^23^. However, in studies conducted in the USA on *I. scapularis*, co-infections rates of *B. burgdorferi* s.s. and *B. microti* were reported to be 83% higher than expected^73^.

As a possible explanation, the authors suggested lower resistance and/or higher tolerance of small mammals to these pathogens leading to correlated reservoir competence or facilitation of transmission or proliferation of other pathogens in co-infected hosts.

Similar correlation was also found for *Borrelia* spp. and *N. mikurensis* co-infections – *N. mikurensis* prevalence was significantly higher in *Borrelia* spp. – positive ticks compared to *Borrelia* spp. – negative. A recent study in the Netherlands also found a strong positive association between these two pathogens^6^. However, we did not observe a significant effect of co-infection on pathogen loads in ticks. Comparable result of pathogen quantification in questing *I. ricinus* ticks was obtained in the Netherlands^6^. The most possible explanation of this correlation is that most of the co-infections reported in our study were between *B. afzelii* and *N. mikurensis*, which share common reservoir hosts, mainly rodents, thus simultaneous transmission to ticks of these pathogens during bloodmeal was possible^74^. Nevertheless, in other studies a rate of co-infection with *N. mikurensis* and *B. azfelii* in bank voles in Sweden was found to be higher than expected to random co-occurrence which may suggest it could be a result of positive interactions between these pathogens. The possible mechanism of this facilitation is immunosuppression caused by one pathogen, which could favor infection of a second pathogen^75^. In ticks, however, the possible mechanism for such a positive interaction has not yet been investigated.

Interestingly, we found a positive correlation of co-infection with *N. mikurensis* and *B. microti* and the prevalences of these pathogens. As these pathogens also share a common host reservoir, co-transmission is possible during a single feeding^23,74^. However, loads of *N. mikurensis* were twice as high in *Babesia* spp. infected ticks compared to ticks free of this pathogen, which suggests the possible survival advantage for both pathogens. To the best of our knowledge, no similar results have been obtained to date.

In our study, we did not find any significant correlation between *Borrelia* spp. and *Rickettsia* spp., both in prevalences and loads of these pathogens. Similarly, in studies conducted in Germany, no positive association between these pathogens was found in GLMM analysis^76^. Opposing outcomes, however were obtained in recent studies conducted in the Netherlands, showing a positive association between *B. burgdorferi* s.l. and *R. helvetica* in *I. ricinus*^6^. It should be noted, however, that this relationship was less pronounced than that found between *B. burgdorferi* s.l. and *N. mikurensis* and was not detected in all studied locations.

We also found no significant correlation between *Borrelia burgdorferi* s.l. infection status and loads of other TBPs such as *A. phagocytophilum*, *Babesia* spp. and *Bartonella* spp. in tested ticks. It should be noted that, despite the large quantity of ticks examined, the number of specimens infected with these TBPs was quite low and may not have been sufficient to obtain statistically significant results. The low prevalence of some TBPs in tested ticks is a main limitation of our study, therefore further studies on a larger number of ticks may be required.

## Conclusions

In conclusion, by testing each tick individually and employing the new ddPCR method for quantitative analysis, we were able to asess with high precision the impact of co-infections on both the prevalence and pathogen loads in ticks. We performed one of the first studies linking the pathogen infection rates in ticks feeding on humans with co-infection patterns and potential interactions between tick-borne pathogens coexisting within the arthropod vector. Inter-pathogen relationships can potentially affect the ability of ticks to transmit pathogens or influence the success of pathogen transmission from tick to human, which has great importance from the medical point of view. Understanding the mechanisms by which pathogenic microorganisms and parasites coexist within ticks may be crucial to effective risk prediction not only of the most commonly diagnosed tick-borne disease in Europe as Lyme borreliosis, but also of infections caused by newly emerging tick-borne pathogens.

## Electronic supplementary material

Below is the link to the electronic supplementary material.

Supplementary Material 1

## Data Availability

The datasets used and analysed during this study are available from the corresponding author (JK) on reasonable request. The new nucleotide sequences have been deposited in the GenBank database under accession numbers: PV124223, PV124288 – PV124290, PV173311, PV173333 – PV173342.
