# Supplementary material for: Coexistence of Borrelia spp. with different tick-borne pathogens in Ixodes ricinus ticks removed from humans in Poland
Source: Sci Rep. 2025 Jul 1;15:21684. doi: 10.1038/s41598-025-05885-2 (PMC12216539; doi:10.1038/s41598-025-05885-2)
Supplement: Supplementary file 1 — Supplementary Material 1 [file 41598_2025_5885_MOESM1_ESM.docx]

**Supplementary File 1.** **TBPs distribution in *I. ricinus* ticks removed from humans between 2021-2022.**

| Collection year | No. of tested ticks (N) | No. of positive ticks [%]^a^ | | | | | |
| --- | --- | --- | --- | --- | --- | --- | --- |
|  |  | *Rickettsia* | *Borrelia* | *A.phag* | *Babesia* | *N.mik*^b^ | *Bartonella* |
| 2021 | 835 | 196 (23.5) [20.7- 26.4] | 166 (19.9) [17.3 – 22.7] | 24 (2.9) [1.9 – 4.2] | 23 (2.8) [1.8 – 4.0] | 56 (6.7) [5.2 – 8.6] | 2 (0.2) [0.0 – 0.8] |
| 2022 | 1238 | 151 (12.2) [10.5 – 14.1] | 158 (12.8) [11.0 – 14.7] | 17 (1.4) [0.8 - 2.1] | 11 (0.9) [0.5 – 1.5] | nd | 4 (0.3) [0.1 – 0.8] |
| Total | 2073 | 347 (16.7) [15.2 – 18.4] | 324 (15.6)  [4.1 – 17.2%] | 41 (2.0) [1.4 – 2.6] | 34 (1.6) [1.2 – 2.3] | 56 (6.7) [5.2 – 8.6] | 6 (0.3) [0.1 – 0.6] |

*N. mik* - *N. mikurensis*, *A. phag* - *A. phagocytophilum*.

^a^ Prevalence of positive ticks are given as the percentage with the 95% CI in parentheses [%].

^b^ Only ticks collected in 2021 were tested for presence of *N. mikurensis* DNA (N=832).

**Supplementary File 2. TBPs distribution in *I. ricinus* ticks removed from humans between developmental stages.**

| Tick stage | No. of tested ticks (N) | No. of positive ticks [%]^a^ | | | | | |
| --- | --- | --- | --- | --- | --- | --- | --- |
|  |  | *Rickettsia* | *Borrelia* | *A.phag* | *Babesia* | *N.mik*^b^ | *Bartonella* |
| Larvae | 89 | 11 (12.4) [6.7 – 20.4] | 3 (3.4) [1.0 – 8.7] | 0 (0.0) | 0 (0.0) | 2 (5.3) [1.1 – 15.8] | 0 (0.0) |
| Nymphs | 1447 | 230 (15.9) [14.1 – 17.8] | 212 (14.7) [12.9 – 16.5] | 23 (1.6) [1.0 – 2.3] | 26 (1.8) [1.2 – 2.6] | 35 (6.5) [4.6 – 8.8] | 5 (0.3) [0.1 – 0.8] |
| Adults | 537 | 106 (19.7) [16.5 – 23.3] | 109 (20.3) [17.1 – 23.9] | 18 (3.4) [2.1 – 5.1] | 8 (1.5) [0.7 – 2.8] | 19 (7.5) [4.8 – 11.3] | 1 (0.2) [0.0 – 0.9] |
| Total | 2073 | 347 (16.7) [15.2 – 18.4] | 324 (15.6)  [4.1 – 17.2] | 41 (2.0) [1.4 – 2.6] | 34 (1.6) [1.2 – 2.3] | 56 (6.7)  [5.2 – 8.6] | 6 (0.3) [0.1 – 0.6] |

*N. mik - N. mikurensis, A. phag - A. phagocytophilum.*

^a^ Prevalence of positive ticks are given as the percentage with the 95% CI in parentheses [%].

^b^ Only ticks collected in 2021 were tested for presence of *N. mikurensis* DNA: larvae = 38,

nymphs = 542, adults = 252 (N=832).

**Supplementary File 3. Dual co-infections in individual *Ixodes ricinus* ticks removed from humans in Poland.**

| **Tick stage/no. of specimens** | | **Total no. Ticks** | **Species of co-infecting pathogens** | | |
| --- | --- | --- | --- | --- | --- |
| Nymph | 3 | 5 | *Borrelia afzelii* | + | *Babesia microti* |
| Female | 2 |  |  |  |  |
| Nymph | 1 | 3 | *Borrelia afzelii* | + | *Anaplasma phagocytophilum* |
| Female | 2 |  |  |  |  |
| Larva | 1 | 9 | *Borrelia afzelii* | + | *Neoehrlichia mikurensis* |
| Nymph | 5 |  |  |  |  |
| Female | 2 |  |  |  |  |
| Male | 1 |  |  |  |  |
| Nymph | 2 | 2 | *Borrelia afzelii* | + | *Bartonela taylorii* |
| Nymph | 17 | 25 | *Borrelia afzelii* | + | *Rickettsia* sp. |
| Female | 8 |  |  |  |  |
| Nymph | 4 | 5 | *Borrelia burgdorferii* s.s. | + | *Rickettsia* sp. |
| Female | 1 |  |  |  |  |
| Nymph | 2 | 2 | *Borrelia garinii* | + | *Babesia* sp. |
| Nymph | 8 | 10 | *Borrelia garinii* | + | *Rickettsia* sp. |
| Female | 2 |  |  |  |  |
| Nymph | 1 | 1 | *Borrelia lusitaniae* | + | *Rickettsia* sp. |
| Female | 1 | 1 | *Borrelia spielmanii* | + | *Anaplasma phagocytophilum* |
| Nymph | 2 | 2 | *Borrelia valaisiana* | + | *Rickettsia* sp. |
| Nymph | 1 | 2 | *Borrelia miyamotoi* | + | *Neoehrlichia mikurensis* |
| Female | 1 |  |  |  |  |
| Male | 1 | 1 | *Borrelia miyamotoi* | + | *Rickettsia* sp. |
| Nymph | 5 | 7 | *Borrelia* sp. | + | *Rickettsia* sp. |
| Female | 2 |  |  |  |  |
| Nymph | 1 | 1 | *Borrelia* sp. | + | *Neoehrlichia mikurensis* |
| Nymph | 4 | 7 | *Rickettsia* sp. | + | *Anaplasma phagocytophilum* |
| Female | 3 |  |  |  |  |
| Nymph | 5 | 6 | *Rickettsia* sp. | + | *Neoehrlichia mikurensis* |
| Female | 1 |  |  |  |  |
| Nymph | 1 | 1 | *Rickettsia* sp. | + | *Babesia microti* |
| Nymph | 1 | 1 | *Rickettsia* sp. | + | *Babesia venatorum* |
| Nymph | 1 | 1 | *Rickettsia* sp. | + | *Babesia* sp. |
| Nymph | 1 | 3 | *Babesia microti* | + | *Neoehrlichia mikurensis* |
| Female | 1 |  |  |  |  |
| Male | 1 |  |  |  |  |
| Female | 1 | 1 | *Babesia venatorum* | + | *Bartonela taylorii* |

**Supplementary File 4. Triple co-infections in individual *Ixodes ricinus* ticks removed from humans in Poland.**

| **Tick stage/no. of specimens** | | **Species of co-infecting pathogens** | | | | |
| --- | --- | --- | --- | --- | --- | --- |
| Nymph | 2 | *Borrelia afzelii* | + | *Babesia microti* | + | *Neoehrlichia mikurensis* |
| Female | 1 | *Borrelia afzelii* | + | *Babesia microti* | + | *Rickettsia* sp. |
| Nymph | 1 | *Borrelia afzelii* | + | *Babesia* sp. | + | *Rickettsia* sp. |
| Female | 1 | *Borrelia afzelii* | + | *Anaplasma phagocytophilum* | + | *Rickettsia* sp. |
| Female | 2 | *Borrelia burgdorferi s.s.* | + | *Borrelia miyamotoi* | + | *Rickettsia* sp. |
| Nymph | 3 | *Borrelia sp.* | + | *Neoehrlichia mikurensis* | + | *Rickettsia* sp. |

**Supplementary file 5. Detailed results of quantitive analyses regarding loads of selected TBPs depending on infection status of ticks removed from humans**

| **Infection status of ticks^c^** | **Pathogen loads in ticks** | | | | | | | | | | | | | | |
| --- | --- | --- | --- | --- | --- | --- | --- | --- | --- | --- | --- | --- | --- | --- | --- |
|  | ***Borrelia*** | | | ***Rickettsia*** | | | ***N. mikurensis*** | | | ***A. phagocytophilum*** | | | ***Babesia*** | | |
|  | **N^b^** | **Targets/μL^a^** | **P value** | **N^b^** | **Targets/μL^a^** | **P value** | **N^b^** | **Targets/μL^a^** | **P value** | **N^b^** | **Targets/μL^a^** | **P value** | **N^b^** | **Targets/μL^a^** | **P value** |
| ***Bor* – neg** |  |  |  | 184 | 581.6 [310.5 – 852.6] | 0.283 | 184 | 179.0 [39.5 – 318.4] | 0.190 | 184 | 476.8 [96.4 – 857.3] | 0.643 | 184 | 128.1 [28.6 – 227.6] | 0.961 |
| ***Bor* – pos** |  |  |  | 22 | 784.3 [429.5 – 1139.0] |  | 9 | 239.9  [-43.6 – 523.5] |  | 3 | 124.9 [-110.4 – 360.2] |  | 8 | 48.9 [-0.1 – 97.9] |  |
| ***Rick* – neg** | 184 | 31.7 [24.5 – 38.9] | 0.106 |  |  |  |  |  |  |  |  |  |  |  |  |
| ***Rick* – pos** | 42 | 44.8 [25.3 – 64.4] |  |  |  |  |  |  |  |  |  |  |  |  |  |
| ***N. mik* – neg** | 122 | 44.2 [25.2 – 63.3] | 0.884 |  |  |  |  |  |  |  |  |  | 16 | 60.5 [-12.0 – 133.0] | 0.026* |
| ***N. mik* – pos** | 9 | 38.5 [1.3 – 75.8] |  |  |  |  |  |  |  |  |  |  | 5 | 488.7 [-181.9 – 1159.2] |  |
| ***A. phag* – neg** | 227 | 47.2 [34.3 – 60.2] | 0.801 |  |  |  |  |  |  |  |  |  |  |  |  |
| ***A. phag* – pos** | 5 | 53.4 [-41.2 – 148.1] |  |  |  |  |  |  |  |  |  |  |  |  |  |
| ***Bab* – neg** | 221 | 47.5 [34.2 – 60.8 ] | 0.749 |  |  |  | 31 | 188.6 [52.5 – 324.8] | 0.178 |  |  |  |  |  |  |
| ***Bab* – pos** | 11 | 44.2 [6.5 – 81.9] |  |  |  |  | 4 | 330.1 [-192.3 – 853.5] |  |  |  |  |  |  |  |

^a^ The concentrations of target DNA copies in ticks are presented as copies of template per μL of the ﬁnal 1 × ddPCR reaction with the 95% CI in parentheses

^b^ Number of tested of tested ticks

^c^ neg - negative, pos - positive, Bor - Borrelia burgdorferi s.l., Rick - Rickettsia, N. mik - N. mikurensis, A. phag - A. phagocytophilum, Bab - Babesia

An asterisk indicates a P<0.05 determined by Mann-Whitney U-test.
